# *N*-(2-Methyl­phen­yl)succinamic acid

**DOI:** 10.1107/S1600536810010329

**Published:** 2010-03-24

**Authors:** B. Thimme Gowda, Sabine Foro, B. S. Saraswathi, Hartmut Fuess

**Affiliations:** a Department of Chemistry, Mangalore University, Mangalagangotri 574 199, Mangalore, India; b Institute of Materials Science, Darmstadt University of Technology, Petersenstrasse 23, D-64287 Darmstadt, Germany

## Abstract

In the crystal structure of the title compound, C_11_H_13_NO_3_, the conformations of the N—H and C=O bonds in the amide segment are *anti* to each other and that of the amide H atom is *syn* to the *ortho*-methyl group in the benzene ring. In the crystal, O—H⋯O interactions lead to carboxylic acid inversion dimers and inter­molecular N—H⋯O hydrogen bonds link the mol­ecules into infinite chains. In addition, the crystal structure exhibits inter­molecular C—H⋯π inter­actions between one of the methyl H atoms and the benzene ring of neighbouring mol­ecules.

## Related literature

For our study of the effect of ring and side-chain substitutions on the crystal structures of anilides and for related structures, see: Gowda *et al.* (2007 ▶; 2009 ▶; 2010 ▶); Jagannathan *et al.* (1994 ▶). For the modes of inter­linking carboxylic acids by hydrogen bonds, see: Leiserowitz (1976 ▶).
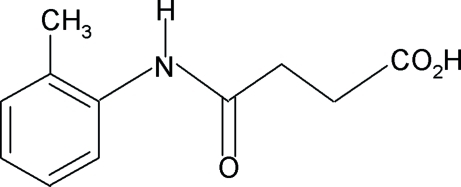


## Experimental

### 

#### Crystal data

C_11_H_13_NO_3_
*M* *_r_* = 207.22
Triclinic, 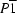

*a* = 4.7756 (9) Å
*b* = 6.1854 (9) Å
*c* = 18.275 (3) Å
α = 86.20 (2)°
β = 83.02 (1)°
γ = 88.45 (2)°
*V* = 534.55 (15) Å^3^
*Z* = 2
Mo *K*α radiation
μ = 0.09 mm^−1^
*T* = 299 K
0.40 × 0.12 × 0.06 mm

#### Data collection

Oxford Diffraction Xcalibur diffractometer
Absorption correction: multi-scan (*CrysAlis RED*; Oxford Diffraction, 2009 ▶) *T*_min_ = 0.963, *T*_max_ = 0.994
2928 measured reflections
1873 independent reflections
1426 reflections with *I* > 2σ(*I*)
*R*_int_ = 0.010

#### Refinement

*R*[*F* ^2^ > 2σ(*F* ^2^)] = 0.060
*wR*(*F* ^2^) = 0.149
*S* = 1.10
1873 reflections
143 parameters
13 restraints
H atoms treated by a mixture of independent and constrained refinement
Δρ_max_ = 0.33 e Å^−3^
Δρ_min_ = −0.31 e Å^−3^

### 

Data collection: *CrysAlis CCD* (Oxford Diffraction, 2009 ▶); cell refinement: *CrysAlis RED* (Oxford Diffraction, 2009 ▶); data reduction: *CrysAlis RED*; program(s) used to solve structure: *SHELXS97* (Sheldrick, 2008 ▶); program(s) used to refine structure: *SHELXL97* (Sheldrick, 2008 ▶); molecular graphics: *PLATON* (Spek, 2009 ▶) and *DIAMOND* (Brandenburg, 1998 ▶); software used to prepare material for publication: *SHELXL97*.

## Supplementary Material

Crystal structure: contains datablocks I, global. DOI: 10.1107/S1600536810010329/lx2138sup1.cif

Structure factors: contains datablocks I. DOI: 10.1107/S1600536810010329/lx2138Isup2.hkl

Additional supplementary materials:  crystallographic information; 3D view; checkCIF report

## Figures and Tables

**Table 1:** Hydrogen-bond geometry (Å, °) *Cg* is the centroid of the C1–C6 ring.

*D*—H⋯*A*	*D*—H	H⋯*A*	*D*⋯*A*	*D*—H⋯*A*
N1—H1*N*⋯O1^i^	0.81 (4)	2.13 (4)	2.922 (3)	164 (3)
O3—H3*O*⋯O2^ii^	0.85 (2)	1.82 (2)	2.664 (3)	173 (5)
C11—H11*A*⋯*Cg*^iii^	0.96	2.81	3.596 (4)	139

Symmetry codes: (i) 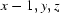; (ii) 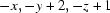; (iii) 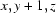.

**Fig. 1.**
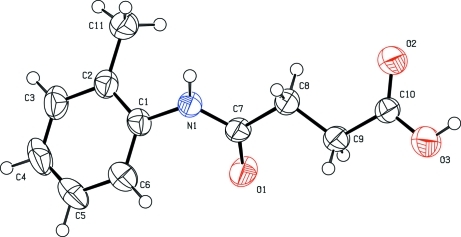
Molecular structure of the title compound, showing the atom labelling scheme. The displacement ellipsoids are drawn at the 50% probability level. The H atoms are represented as small spheres of arbitrary radii.

**Fig. 2.**
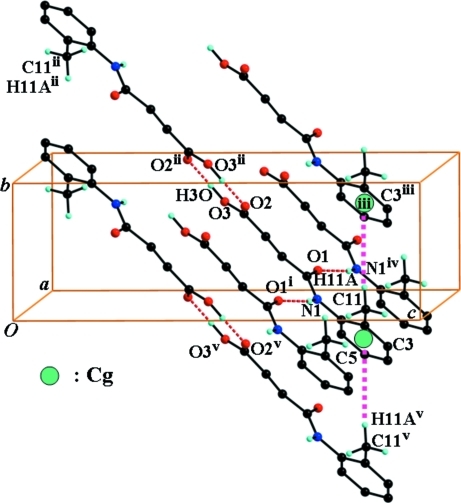
O—H···O, N—H···O and C—H···π interactions (dotted lines) in the crystal structure of the title compound. Cg denotes the ring centroid. [Symmetry codes : (i) x - 1, y, z; (ii) - x, - y + 2, - z + 1; (iii) x, y + 1, z; (iv) x + 1, y, z; (v) x, y - 1, z.]

## supplementary crystallographic information

### Comment

As a part of studying the effect of ring and side chain substitutions on the crystal structures of anilides (Gowda *et al.*, 2007; 2009; 2010), the crystal structure of *N*-(2-methylphenyl)succinamic acid (I) has been determined. The conformations of N—H and C═O bonds in the amide segment are *anti* to each other. The conformation of the amide oxygen and the carbonyl oxygen of the acid segment are also *anti* to each other, similar to that observed in *N*-(2-chlorophenyl)succinamic acid (II) (Gowda *et al.*, 2009), but contrary to the *syn* conformation observed in *N*-(3-methylphenyl)succinamic acid (III) (Gowda *et al.*, 2010). Further, the conformation of both the C═O bonds are *anti* to the H atoms of their adjacent –CH_2_ groups (Fig. 1) and the C═O and O—H bonds of the acid group are in *syn* position to each other, similar to that observed in (II) and (III).

The conformation of the amide hydrogen is *syn* to the *ortho*- methyl group in the benzene ring, similar to that observed between the amide hydrogen and the *ortho*-Cl in (II), but contrary to the *anti* conformation observed between the amide hydrogen and the *meta*-methyl group in the benzene ring of (III). The intermolecular O—H···O and N—H···O hydrogen bonds pack the molecules into infinite chains in the structure (Table 1, Fig.2). Additionally, the crystal packing (Fig. 2) is further stabilized by intermolecular C—H···π interactions between the methyl H atom and the benzene ring of neighbouring molecules, with a C11—H11A···Cg^iii^ (Table 1; Cg is the centroid of the C1–C6 benzene ring). The modes of interlinking carboxylic acids by hydrogen bonds is described elsewhere (Leiserowitz, 1976). The packing of molecules involving dimeric hydrogen bonded association of each carboxyl group with a centrosymmetrically related neighbor has also been observed (Jagannathan *et al.*, 1994).

### Experimental

The solution of succinic anhydride (0.01 mole) in toluene (25 ml) was treated dropwise with the solution of *o*-toluidine (0.01 mole) also in toluene (20 ml) with constant stirring. The resulting mixture was stirred for about one h and set aside for an additional hour at room temperature for completion of the reaction. The mixture was then treated with dilute hydrochloric acid to remove the unreacted *o*-toluidine. The resultant solid *N*-(2-methylphenyl)-succinamic acid was filtered under suction and washed thoroughly with water to remove the unreacted succinic anhydride and succinic acid. It was recrystallized to constant melting point from ethanol. The purity of the compound was checked by elemental analysis and characterized by its infrared and NMR spectra. The needle like colorless single crystals used in X-ray diffraction studies were grown in ethanolic solution by slow evaporation at room temperature.

### Refinement

The H atom of the NH group was located in a difference map and its position refined with N—H = 0.82 (3) %A. The H atom of the OH group was located in a difference map and later restrained to the distance O—H = 0.82 (2) Å. The other H atoms were positioned with idealized geometry using a riding model with C—H = 0.93–0.97 Å. All H atoms were refined with isotropic displacement parameters (set to 1.2 times of the *U*_eq_ of the parent atom). O2 and O3 are slightly disordered and their U^ij^ components were restrained to approximate isotropic behavoir.

### Crystal data

**Table:**

C_11_H_13_NO_3_	*Z* = 2
*M**_r_* = 207.22	*F*(000) = 220
Triclinic, *P*1	*D*_x_ = 1.287 Mg m^−^^3^
Hall symbol: -P 1	Mo *K*α radiation, λ = 0.71073 Å
*a* = 4.7756 (9) Å	Cell parameters from 1326 reflections
*b* = 6.1854 (9) Å	θ = 3.3–27.7°
*c* = 18.275 (3) Å	µ = 0.09 mm^−^^1^
α = 86.20 (2)°	*T* = 299 K
β = 83.02 (1)°	Needle, colourless
γ = 88.45 (2)°	0.40 × 0.12 × 0.06 mm
*V* = 534.55 (15) Å^3^	

### Data collection

**Table:**

Oxford Diffraction Xcalibur diffractometer	1873 independent reflections
Radiation source: fine-focus sealed tube	1426 reflections with *I* > 2σ(*I*)
graphite	*R*_int_ = 0.010
Detector resolution: 10.0 pixels mm^-1^	θ_max_ = 25.0°, θ_min_ = 3.3°
φ and ω scans	*h* = −5→4
Absorption correction: multi-scan (*CrysAlis RED*; Oxford Diffraction, 2009)	*k* = −7→7
*T*_min_ = 0.963, *T*_max_ = 0.994	*l* = −21→21
2928 measured reflections	

### Refinement

**Table:**

Refinement on *F*^2^	Primary atom site location: structure-invariant direct methods
Least-squares matrix: full	Secondary atom site location: difference Fourier map
*R*[*F*^2^ > 2σ(*F*^2^)] = 0.060	Hydrogen site location: difference Fourier map
*wR*(*F*^2^) = 0.149	H atoms treated by a mixture of independent and constrained refinement
*S* = 1.10	*w* = 1/[σ^2^(*F*_o_^2^) + (0.0463*P*)^2^ + 0.4371*P*] where *P* = (*F*_o_^2^ + 2*F*_c_^2^)/3
1873 reflections	(Δ/σ)_max_ < 0.001
143 parameters	Δρ_max_ = 0.33 e Å^−^^3^
13 restraints	Δρ_min_ = −0.31 e Å^−^^3^

### Special details

**Table:**

Geometry. All esds (except the esd in the dihedral angle between two l.s. planes) are estimated using the full covariance matrix. The cell esds are taken into account individually in the estimation of esds in distances, angles and torsion angles; correlations between esds in cell parameters are only used when they are defined by crystal symmetry. An approximate (isotropic) treatment of cell esds is used for estimating esds involving l.s. planes.
Refinement. Refinement of F^2^ against ALL reflections. The weighted R-factor wR and goodness of fit S are based on F^2^, conventional R-factors R are based on F, with F set to zero for negative F^2^. The threshold expression of F^2^ > 2sigma(F^2^) is used only for calculating R-factors(gt) etc. and is not relevant to the choice of reflections for refinement. R-factors based on F^2^ are statistically about twice as large as those based on F, and R- factors based on ALL data will be even larger.

### Fractional atomic coordinates and isotropic or equivalent isotropic displacement parameters (Å^2^)

**Table:**

	*x*	*y*	*z*	*U*_iso_*/*U*_eq_	
C1	0.0519 (5)	−0.0093 (4)	0.80080 (16)	0.0415 (7)	
C2	−0.0898 (6)	−0.0197 (5)	0.87207 (16)	0.0445 (7)	
C3	−0.0147 (7)	−0.1877 (5)	0.92098 (19)	0.0577 (9)	
H3	−0.1085	−0.1994	0.9687	0.069*	
C4	0.1940 (8)	−0.3363 (5)	0.9006 (2)	0.0685 (10)	
H4	0.2402	−0.4465	0.9344	0.082*	
C5	0.3342 (8)	−0.3227 (5)	0.8307 (2)	0.0648 (10)	
H5	0.4767	−0.4227	0.8170	0.078*	
C6	0.2631 (7)	−0.1594 (5)	0.78027 (19)	0.0538 (8)	
H6	0.3572	−0.1504	0.7326	0.065*	
C7	0.1616 (5)	0.2852 (5)	0.70612 (15)	0.0424 (7)	
C8	0.0310 (5)	0.4576 (5)	0.65761 (16)	0.0476 (8)	
H8A	−0.1193	0.3942	0.6354	0.057*	
H8B	−0.0521	0.5710	0.6880	0.057*	
C9	0.2412 (6)	0.5563 (5)	0.59738 (17)	0.0504 (8)	
H9A	0.4044	0.6001	0.6192	0.060*	
H9B	0.3040	0.4467	0.5632	0.060*	
C10	0.1294 (6)	0.7472 (5)	0.55537 (16)	0.0474 (8)	
C11	−0.3113 (7)	0.1471 (6)	0.89617 (19)	0.0583 (9)	
H11A	−0.2352	0.2894	0.8858	0.087*	
H11B	−0.3683	0.1241	0.9483	0.087*	
H11C	−0.4716	0.1340	0.8699	0.087*	
N1	−0.0225 (5)	0.1572 (4)	0.74871 (14)	0.0440 (6)	
H1N	−0.189 (7)	0.181 (6)	0.7458 (19)	0.066*	
O1	0.4164 (4)	0.2708 (4)	0.70798 (13)	0.0665 (8)	
O2	−0.0821 (6)	0.8479 (5)	0.57850 (15)	0.0915 (10)	
O3	0.2743 (7)	0.8021 (5)	0.49406 (15)	0.0937 (10)	
H3O	0.201 (10)	0.914 (6)	0.474 (3)	0.141*	

### Atomic displacement parameters (Å^2^)

**Table:**

	*U*^11^	*U*^22^	*U*^33^	*U*^12^	*U*^13^	*U*^23^
C1	0.0352 (14)	0.0405 (15)	0.0501 (17)	−0.0014 (12)	−0.0162 (12)	0.0080 (13)
C2	0.0374 (15)	0.0466 (17)	0.0493 (17)	−0.0046 (12)	−0.0111 (13)	0.0108 (14)
C3	0.0569 (19)	0.056 (2)	0.058 (2)	−0.0059 (16)	−0.0119 (15)	0.0210 (16)
C4	0.078 (2)	0.0465 (19)	0.081 (3)	0.0029 (18)	−0.028 (2)	0.0240 (18)
C5	0.068 (2)	0.0458 (19)	0.082 (3)	0.0179 (16)	−0.0214 (19)	0.0020 (18)
C6	0.0546 (18)	0.0494 (18)	0.059 (2)	0.0056 (15)	−0.0137 (15)	−0.0010 (15)
C7	0.0285 (13)	0.0535 (17)	0.0441 (16)	0.0032 (12)	−0.0069 (11)	0.0090 (13)
C8	0.0322 (14)	0.0579 (19)	0.0505 (18)	0.0028 (13)	−0.0069 (12)	0.0156 (15)
C9	0.0401 (15)	0.0571 (19)	0.0502 (18)	0.0089 (14)	−0.0007 (13)	0.0130 (15)
C10	0.0388 (15)	0.0537 (18)	0.0463 (17)	0.0047 (14)	−0.0009 (13)	0.0122 (14)
C11	0.0491 (18)	0.066 (2)	0.056 (2)	0.0067 (16)	−0.0015 (15)	0.0094 (16)
N1	0.0280 (11)	0.0517 (15)	0.0509 (14)	0.0021 (11)	−0.0099 (10)	0.0143 (11)
O1	0.0273 (10)	0.0858 (17)	0.0817 (17)	0.0013 (10)	−0.0105 (10)	0.0374 (13)
O2	0.0759 (17)	0.0922 (19)	0.0891 (19)	0.0379 (15)	0.0237 (14)	0.0444 (15)
O3	0.102 (2)	0.0900 (19)	0.0713 (17)	0.0412 (16)	0.0292 (15)	0.0383 (14)

### Geometric parameters (Å, °)

**Table:**

C1—C6	1.387 (4)	C7—C8	1.512 (4)
C1—C2	1.391 (4)	C8—C9	1.506 (4)
C1—N1	1.423 (3)	C8—H8A	0.9700
C2—C3	1.393 (4)	C8—H8B	0.9700
C2—C11	1.505 (4)	C9—C10	1.488 (4)
C3—C4	1.372 (5)	C9—H9A	0.9700
C3—H3	0.9300	C9—H9B	0.9700
C4—C5	1.367 (5)	C10—O2	1.218 (4)
C4—H4	0.9300	C10—O3	1.274 (4)
C5—C6	1.385 (5)	C11—H11A	0.9600
C5—H5	0.9300	C11—H11B	0.9600
C6—H6	0.9300	C11—H11C	0.9600
C7—O1	1.222 (3)	N1—H1N	0.81 (4)
C7—N1	1.340 (4)	O3—H3O	0.85 (2)
			
C6—C1—C2	120.7 (3)	C7—C8—H8A	109.0
C6—C1—N1	120.0 (3)	C9—C8—H8B	109.0
C2—C1—N1	119.3 (3)	C7—C8—H8B	109.0
C1—C2—C3	117.5 (3)	H8A—C8—H8B	107.8
C1—C2—C11	121.4 (2)	C10—C9—C8	114.1 (2)
C3—C2—C11	121.1 (3)	C10—C9—H9A	108.7
C4—C3—C2	121.7 (3)	C8—C9—H9A	108.7
C4—C3—H3	119.1	C10—C9—H9B	108.7
C2—C3—H3	119.1	C8—C9—H9B	108.7
C5—C4—C3	120.2 (3)	H9A—C9—H9B	107.6
C5—C4—H4	119.9	O2—C10—O3	122.0 (3)
C3—C4—H4	119.9	O2—C10—C9	122.6 (3)
C4—C5—C6	119.7 (3)	O3—C10—C9	115.4 (3)
C4—C5—H5	120.1	C2—C11—H11A	109.5
C6—C5—H5	120.1	C2—C11—H11B	109.5
C5—C6—C1	120.1 (3)	H11A—C11—H11B	109.5
C5—C6—H6	119.9	C2—C11—H11C	109.5
C1—C6—H6	119.9	H11A—C11—H11C	109.5
O1—C7—N1	123.0 (2)	H11B—C11—H11C	109.5
O1—C7—C8	121.8 (2)	C7—N1—C1	124.8 (2)
N1—C7—C8	115.1 (2)	C7—N1—H1N	117 (3)
C9—C8—C7	112.8 (2)	C1—N1—H1N	118 (3)
C9—C8—H8A	109.0	C10—O3—H3O	110 (4)
			
C6—C1—C2—C3	−1.1 (4)	N1—C1—C6—C5	−179.4 (3)
N1—C1—C2—C3	178.7 (3)	O1—C7—C8—C9	17.9 (5)
C6—C1—C2—C11	177.5 (3)	N1—C7—C8—C9	−164.8 (3)
N1—C1—C2—C11	−2.7 (4)	C7—C8—C9—C10	−171.8 (3)
C1—C2—C3—C4	1.0 (5)	C8—C9—C10—O2	17.7 (5)
C11—C2—C3—C4	−177.6 (3)	C8—C9—C10—O3	−164.0 (3)
C2—C3—C4—C5	−0.2 (5)	O1—C7—N1—C1	0.0 (5)
C3—C4—C5—C6	−0.6 (6)	C8—C7—N1—C1	−177.2 (3)
C4—C5—C6—C1	0.5 (5)	C6—C1—N1—C7	−48.9 (4)
C2—C1—C6—C5	0.4 (5)	C2—C1—N1—C7	131.2 (3)

### Hydrogen-bond geometry (Å, °)

**Table:**

Cg is the centroid of the C1–C6 ring.

**Table:**

*D*—H···*A*	*D*—H	H···*A*	*D*···*A*	*D*—H···*A*
N1—H1N···O1^i^	0.81 (4)	2.13 (4)	2.922 (3)	164 (3)
O3—H3O···O2^ii^	0.85 (2)	1.82 (2)	2.664 (3)	173 (5)
C11—H11A···Cg^iii^	0.96	2.81	3.596 (4)	139

Symmetry codes: (i) *x*−1, *y*, *z*; (ii) −*x*, −*y*+2, −*z*+1; (iii) *x*, *y*+1, *z*.
